# We Make Our Bow

**Published:** 1869-04

**Authors:** 


### THE BISTOURY.

ELMIRA, N. Y., APRIL, 1869.

Thad S. UpDeGraff, M. D., Editor. Volume V.---No. 1.

The BISTOURY is published Quarterly, upon the 1st of April, July, October and January, at Fifty Cents a year in advance.

☞ For Club Rates see Prospectus on inside of Title Page.

### We make our Bow.

Five years ago we began the publication of the BISTOURY, then a little sheet with less than three thousand subscribers. Since then we have enlarged and improved it every year, rendering it more useful and interesting at every issue, until it has reached a circulation of more than twenty thousand copies. Heretofore it has been a free journal, supported by advertisements and our own pocket, our object having been to warn the public against all manner of charlatanism and humbuggery, and to educate the family circle upon medical subjects which we have considered profitable for them to learn.

How well we have succeeded in our aim we leave our readers to judge.

We now offer them the BISTOURY in a much more improved form, and have added several new features to it.

The profession will thank us for offering them the latest MEDICAL ITEMS and NEWS, culled from the standard medical journals of the world, and condensed in a readable and attractive form.

The DOMESTIC MEDICINE and HOUSEKEEPERS DEPARTMENTS will be invaluable to every family, while the articles upon health, education and the sciences will be interesting and instructive to every one laying any claim to intelligence.

We have placed our subscription price at *Fifty Cents a Year*, bringing our journal within the reach of every one who is entitled to be the head of a family. We confidently hope and expect that at least two thirds of those who have received the BISTOURY gratuitously heretofore, will not decline to pay this small sum for our journal in its new dress.

That we can reach a circulation of one hundred thousand copies in one year we have not the least doubt, for our very large cash premiums, offered to those who will interest them selves in extending our circulation, will certainly bring us hosts of subscribers from all over the land. We promise to enlarge our journal and introduce new features for the approbation of the public as rapidly as their subscriptions will allow us so to do. Therefore, the more money we receive, the more we will expend in extending the usefulness of our journal.

We now offer the BISTOURY to the public, hoping that it may meet with a favorable reception, and earnestly desiring that it may be of real service and usefulness in every household to which it may gain admission. And, if at the end of every quarter it shall be eagerly looked for and carefully read, the editor will have the grateful satisfaction of knowing that his work has been appreciated, and will feel repaid accordingly.

